# A novel glycosylation-related gene signature predicts survival in patients with lung adenocarcinoma

**DOI:** 10.1186/s12859-022-05109-8

**Published:** 2022-12-27

**Authors:** Jin-xiao Liang, Qian Chen, Wei Gao, Da Chen, Xin-yu Qian, Jin-qiao Bi, Xing-chen Lin, Bing-bing Han, Jin-shi Liu

**Affiliations:** 1 Department of Oncological Surgery, Cancer Hospital of the University of Chinese Academy of Sciences (Zhejiang Cancer Hospital), No. 1 of Banshan East Road, Hangzhou, 310022 Zhejiang Province Republic of China; 2 Institute of Cancer and Basic Medicine (IBMC), Chinese Academy of Sciences, Hangzhou, People’s Republic of China; 3 School of Medicine, Zhejiang University City College, Hangzhou, People’s Republic of China

**Keywords:** Lung adenocarcinoma (LUAD), Glycosyltransferases (GTs), Prognosis, Immune cells

## Abstract

**Background:**

Lung adenocarcinoma (LUAD) is the most common malignant tumor that seriously affects human health. Previous studies have indicated that abnormal levels of glycosylation promote progression and poor prognosis of lung cancer. Thus, the present study aimed to explore the prognostic signature related to glycosyltransferases (GTs) for LUAD.

**Methods:**

The gene expression profiles were obtained from The Cancer Genome Atlas (TCGA) database, and GTs were obtained from the GlycomeDB database. Differentially expressed GTs-related genes (DGTs) were identified using edge package and Venn diagram. Gene Ontology (GO), Kyoto Encyclopedia of Genes and Genomes (KEGG), and ingenuity pathway analysis (IPA) methods were used to investigate the biological processes of DGTs. Subsequently, Cox and Least Absolute Shrinkage and Selection Operator (LASSO) regression analyses were performed to construct a prognostic model for LUAD. Kaplan–Meier (K–M) analysis was adopted to explore the overall survival (OS) of LUAD patients. The accuracy and specificity of the prognostic model were evaluated by receiver operating characteristic analysis (ROC). In addition, single-sample gene set enrichment analysis (ssGSEA) algorithm was used to analyze the infiltrating immune cells in the tumor environment.

**Results:**

A total of 48 DGTs were mainly enriched in the processes of glycosylation, glycoprotein biosynthetic process, glycosphingolipid biosynthesis-lacto and neolacto series, and cell-mediated immune response. Furthermore, *B3GNT3*, *MFNG*, *GYLTL1B*, *ALG3*, and *GALNT13* were screened as prognostic genes to construct a risk model for LUAD, and the LUAD patients were divided into high- and low-risk groups. K–M curve suggested that patients with a high-risk score had shorter OS than those with a low-risk score. The ROC analysis demonstrated that the risk model efficiently diagnoses LUAD. Additionally, the proportion of infiltrating aDCs (p < 0.05) and Tgds (p < 0.01) was higher in the high-risk group than in the low-risk group. Spearman’s correlation analysis manifested that the prognostic genes (*MFNG* and *ALG3*) were significantly correlated with infiltrating immune cells.

**Conclusion:**

In summary, this study established a novel GTs-related risk model for the prognosis of LUAD patients, providing new therapeutic targets for LUAD. However, the biological role of glycosylation-related genes in LUAD needs to be explored further.

**Supplementary Information:**

The online version contains supplementary material available at 10.1186/s12859-022-05109-8.

## Background

Lung cancer is one of the most common malignant tumors that threaten human health and life quality, and its mortality ranks first among all cancers [1]. Presently, the incidence of lung adenocarcinoma (LUAD) has surpassed that of lung squamous cell carcinoma (LUSC) and has become the most common histological subtype of lung cancer [2]. Early diagnosis and the emergence of new treatments have prolonged the survival of LUAD. However, due to the concealed onset of LUAD, some patients exhibit lymph node metastasis or distant metastasis when screened, and the postoperative recurrence rate was high, which led to a poor prognosis of LUAD. Considering the limitations of LUAD therapy, new therapeutic targets are required to improve the clinical outcomes of the treatment. Hence, a reliable new prognostic model for a feasible targeted therapy is an urgent requisite.

Protein glycosylation is one of the most common post-translational modifications of protein; it is an enzymatic process that forms glycosidic linkages of saccharides with other proteins [3]. The most common protein glycosylation modifications are N-linked and O-linked glycosylation [3]. The glycosylated proteins on the cell surface play critical roles in ligand binding, signal transduction, molecular adhesion, and other cell–cell interactions, which are closely related to major biological processes, such as cell growth, apoptosis, movement, and differentiation [4]. Abnormal glycosylation is a characteristic feature of the malignant transformation of cells and has been proven to be closely associated with a variety of malignant behaviors, such as tumor proliferation, apoptosis, metastasis, and chemotherapy resistance [5]. Immunoregulation is closely related to tumorigenesis and is involved in promoting tumor progression and distant metastasis by triggering inflammation, angiogenesis, and therapeutic response [6]. Glycoproteins are involved in the innate and adaptive immune responses as key molecules that affect the occurrence and development of tumors through endogenous lectins, mutated sphingolipids, and sialic acid domains [7]. Therefore, glycoproteins may become novel biomarkers for treating and predicting LUAD prognosis.

Importantly, protein glycosylation plays a critical role in tumorigenesis and tumor immunity; however, only a relatively few studies have assessed its specific function in LUAD. Thus, in this present study, the bioinformatics analysis of glycosyltransferase (GT), predicted the accuracy of glycosyltransferase as a biomarker in patients with LUAD (Additional file 1).

We screened out the abnormal expression of GTs in LUAD by bioinformatics and established and verified the prognostic model. Interestingly, the prognostic model was closely associated with the prognosis of LUAD and significantly correlated with immune cell infiltration. Thus, this model might provide a theoretical basis for prognosis prediction and immunoanalysis of LUAD in the future, and the GTs involved in the model exhibit significant potential as new therapeutic targets for cancer (Additional file 2).

## Results

### Identification of differentially expressed GTs (DGTs)-related genes

**Fig. 1:**
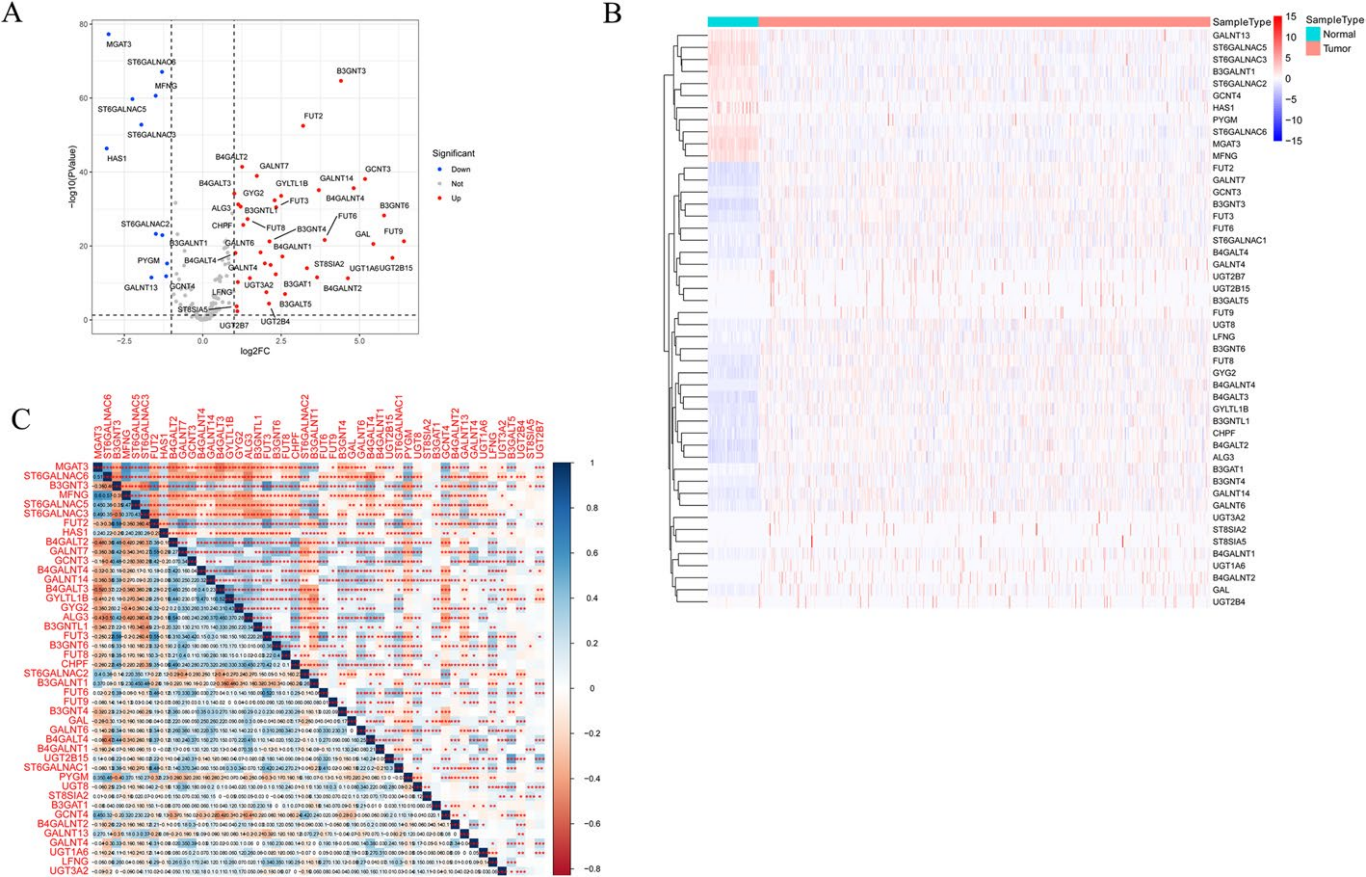
Identification of 48 DGTs. **A** Identification of 48 DGTs. Red dots: up regulation, blue dots: down regulation. **B** Heatmap of the expression of DGTs in normal and LUAD samples from TCGA cohort. **C** The correlations of 48 DGTs were calculated by Pearson analysis. * represents p < 0.05, ** represents p < 0.01, *** represents p < 0.001, blue: positive correlation, red: negative correlation. DGTs: Differentially expressed DGs-related genes

Between the tumor and control samples, we identified 4317 differentially expressed genes (DEGs) through the differentially expressed analysis, including 48 DGTs among 210 GTs obtained from the GlycomeDB database with 37 upregulated and 11 downregulated GTs (Fig. 1A, B; Additional file 4: Table S1). The correlations of the 48 DGTs are displayed in Fig. 1C.

### Functional enrichment analysis

**Fig. 2:**
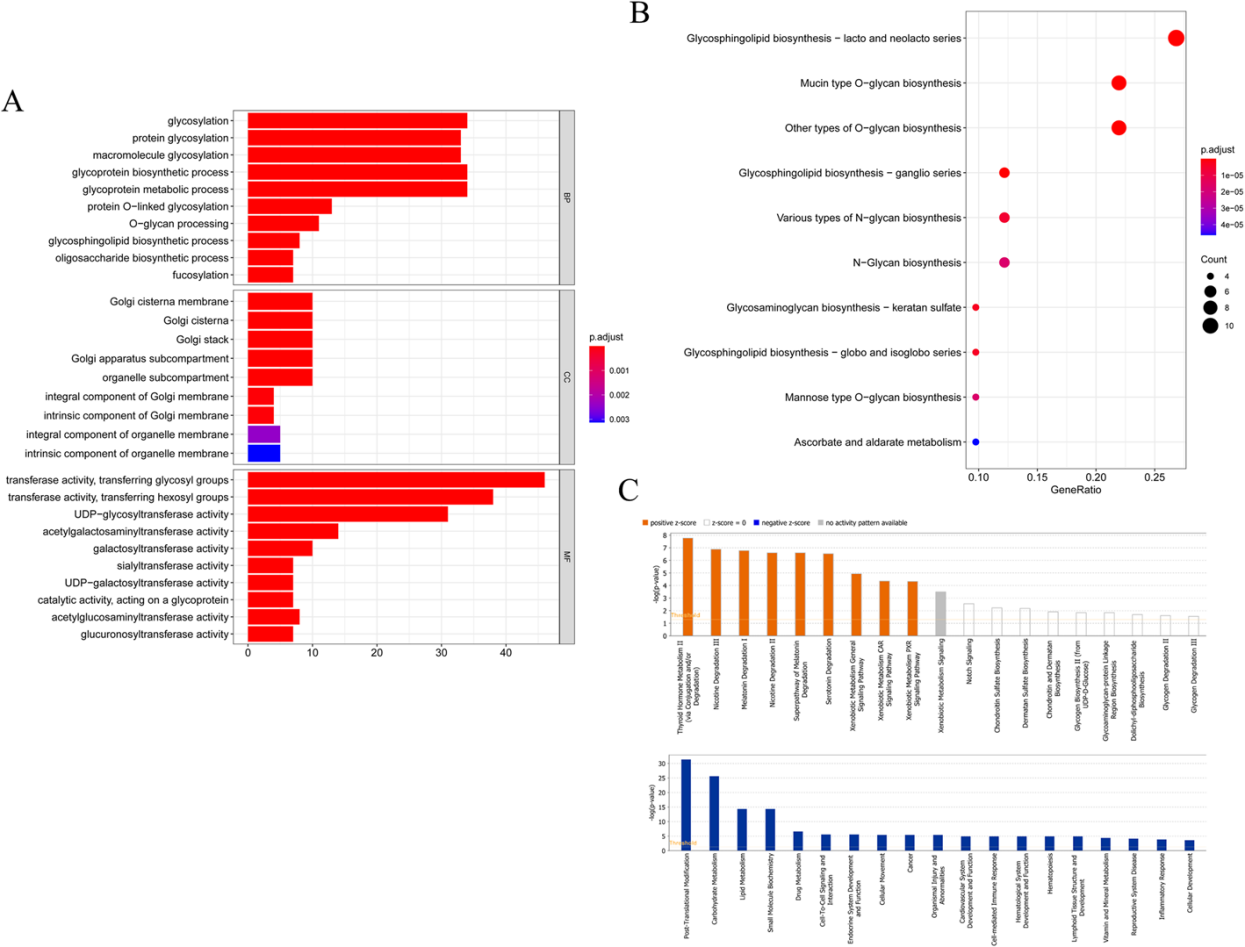
Functional enrichment analysis of DGTs. **A** Bar chart of 29 GO items including 10 biological process (BP) items, 9 cellular components (CC) items, and 10 molecular functions (MF) items. **B** Bubble plot of top 10 KEGG pathways. **C** Histogram of canonical pathways predict by IPA database

The potential functions and pathways of obtained DGTs were detected by Gene Ontology (GO) and Kyoto Encyclopedia of Genes and Genomes (KEGG) analyses. The biological process (BP) results of the GO term indicated that these GTs were mostly enriched in glycosylation, glycoprotein biosynthetic process, and glycoprotein metabolic process. The top cellular components (CC) enrichment terms in GTs were Golgi cisterna membrane, Golgi apparatus subcompartment, and organelle subcompartment. The main molecular functions (MF) involved in GTs were transferase activity, transferring glycosyl groups, transferase activity, and transferring hexosyl groups. GTs were significantly associated with the pathways of glycosphingolipid biosynthesis-lacto and neolacto series and mucin-type O-glycan biosynthesis, as assessed by KEGG analysis (Fig. 2A, B). The Ingenuity Pathway Analysis (IPA) results detected that canonical pathways, thyroid hormone metabolism II (via conjugation and/or degradation), nicotine degradation III, melatonin degradation I, and superpathway of melatonin degradation were statistically significant. Moreover, post-translational modification, carbohydrate metabolism, lipid metabolism, cell-mediated immune response, and inflammatory response were statistically significant (Fig. 2C).

### Development of the risk score model

**Fig. 3:**
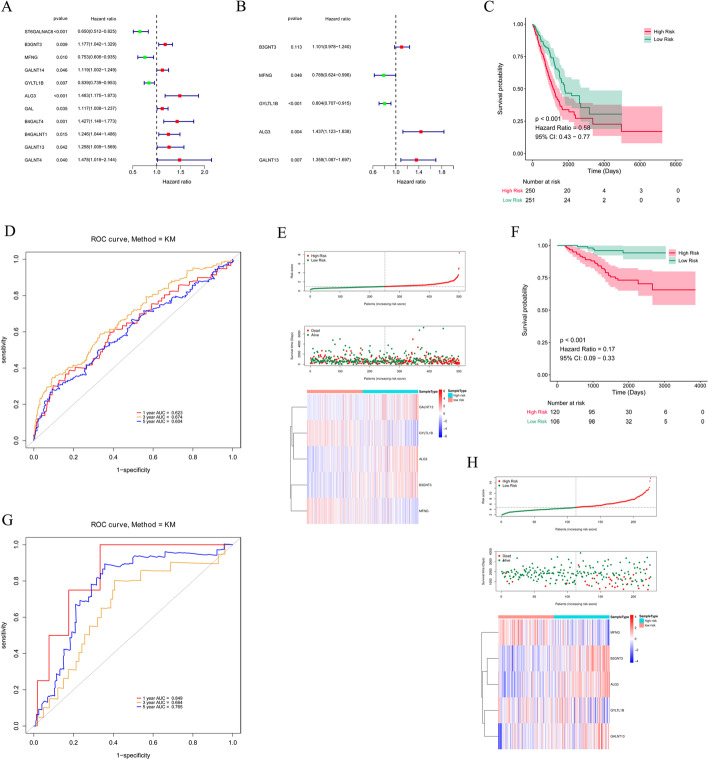
Construction and validation of the prognostic model. **A** Forest plot of the prognostic GTs in LUAD from TCGA cohort by Univariate Cox regression analysis. **B** 5 GTs identified by multivariate. Cox regression analysis. **C** KM survival curve of OS of LUAD patients in the TCGA cohort. **D** ROC curve analysis for measuring the prognostic performance of the signature-based risk score on OS. **E** Risk score, survival status, and 5 GTs expression heatmap of the LUAD patients in the TCGA cohort. **F**–**H** KM survival curve, ROC curve, risk score, survival status, and 5 GTs expression heatmap of LUAD patients in GSE39055 dataset

To evaluate the prognostic value of 48 GTs for LUAD, the Cox regression analysis was conducted in the TCGA database. ST6GALNAC6, B3GNT3, MFNG, GALNT14, GYLTL1B, ALG3, GAL, B4GALT4, B4GALNT1, GALNT13, and GALNT4 were related to the prognosis of LUAD through univariate Cox regression analysis (Fig. 3A). Multivariate Cox regression and stepwise analyses further filtered five candidate prognostic genes to construct a prognostic risk model for LUAD, namely *B3GNT3* [*P* = 0.113, hazard ratio (HR) = 1.101, 95% confidence interval (CI) 0.978–1.240), *MFNG* (*P* = 0.048, HR = 0.789, 95% CI 0.624–0.998), *GYLTL1B* (*P* < 0.001, HR = 0.804, 95% CI 0.707–0.915), *ALG3* (*P* = 0.004, HR = 1.437, 95% CI 1.123–1.838), and *GALNT13* (*P* = 0.007, HR = 1.358, 95% CI 1.087–1.697; Fig. 3B). All patients in the The Cancer Genome Atlas (TCGA) database were classified into high- and low-risk groups by median risk score. The K–M curve analysis demonstrated that patients with high-risk scores (n = 250) showed worse survival overcome than those with low-risk scores (n = 251, *P* < 0.001, HR = 0.58, 95% CI 0.43–0.77, Fig. 3C). We also observed that the area under the curve (AUC) value at 1-, 3-, and 5-years were 0.623, 0.674, and 0.604, respectively, indicating a good performance for the prognostic accuracy of the risk model for LUAD (Fig. 3D). As the risk score increases, the number of deaths in LUAD patients increases. The expression levels of prognostic genes between the two risk groups were illustrated in the heatmap plot (Fig. 3E).

The GSE39055 dataset was used to validate the accuracy of the constructed prognostic model for LUAD. Consistent with the above results, the patients in the low-risk group (n = 106) exhibited a better prognosis compared to those in the high-risk group (n = 120, *P* < 0.001, HR = 0.17, 95% CI 0.09–0.33, Fig. 3F). The receiver operating characteristic (ROC) results also presented an accurate predictive value for survival (1-year AUC = 0.849, 3-year AUC = 0.664, and 5-year AUC = 0.765; Fig. 3G). The risk score distribution, survival status, and prognostic gene expression showed the same trend as the results in the TCGA database (Fig. 3H). In addition, we validated the risk model with the GSE72094 dataset and obtained consistent results (Additional file 3: Fig. S1). In conclusion, the risk model had high accuracy and specificity for LUAD prognosis.

### Correlation between clinical features and risk score

**Fig. 4:**
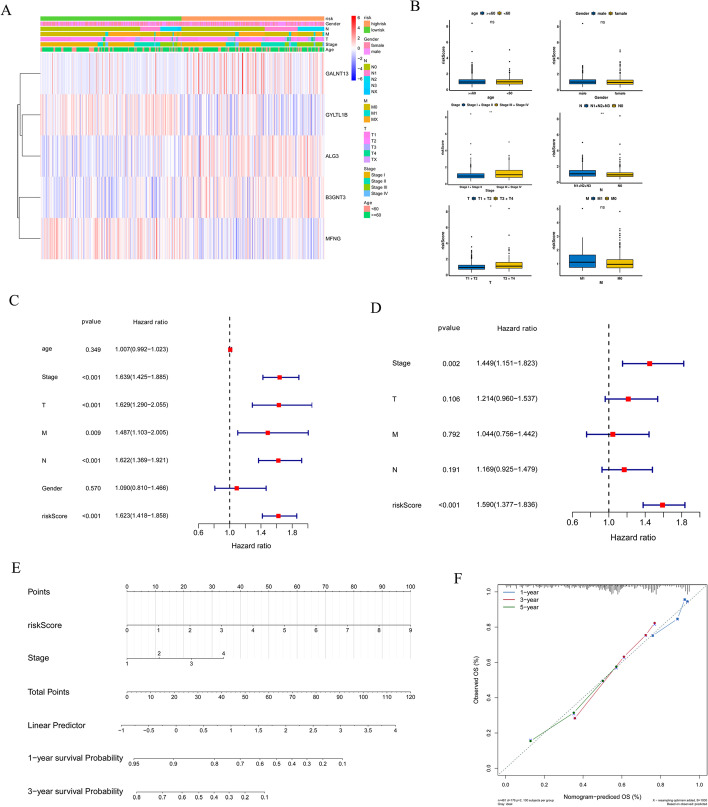
Establishment and evaluation of a nomogram. **A** Heatmap of the five genes between two risk groups and the correlations of the two risk groups and clinical parameters. **B** The relationships between risk score and clinical features. Univariate **C** and multivariate **D** Cox analyses to explore whether risk score was an independent factor in prognosis LUAD. **E** Nomogram based on risk score and Stage. **F** Calibration plots of the nomogram for predicting the probability of 1-, 3- and 5-year survival

Next, we analyzed the associations between the risk signature and age, gender, tumor stage, N stage, T stage, and M stage. As displayed in Fig. 4A, B, the risk score values were significantly associated with stage, N, and T but not age, gender, and M. In addition, univariate and multivariate Cox analyses were used to explore whether the risk score was an independent risk factor for LUAD prognosis. Univariate Cox regression analyses revealed that stage (*P* < 0.001, HR = 1.639, 95% CI 1.452–1.885), T (*P* < 0.001, HR = 1.629, 95% CI 1.290–2.055), M (*P* = 0.009, HR = 1.487, 95% CI 1.103–2.005), N (*P* < 0.001, HR = 1.622, 95% CI 1.369–1.921), and risk score (*P* < 0.001, HR = 1.623, 95% CI 1.418–1.858) were related to the overall survival (OS) of LUAD (Fig. 4C). Among them, stage (*P* = 0.002, HR = 1.449, 95% CI 1.151–1.823) and risk scores (*P* < 0.001, HR = 1.590, 95% CI 1.377–1.836) were ultimately identified to be associated with OS, suggesting that these were independent risk factors for the prognosis of LUAD (Fig. 4D). A nomogram, including the stage and risk score for LUAD was constructed; it exhibited a trend similar to the ideal model, suggesting high reliability for predicting the survival probability of LUAD (Fig. 4E, F).

### Analysis of the immune environment of LUAD

**Fig. 5:**
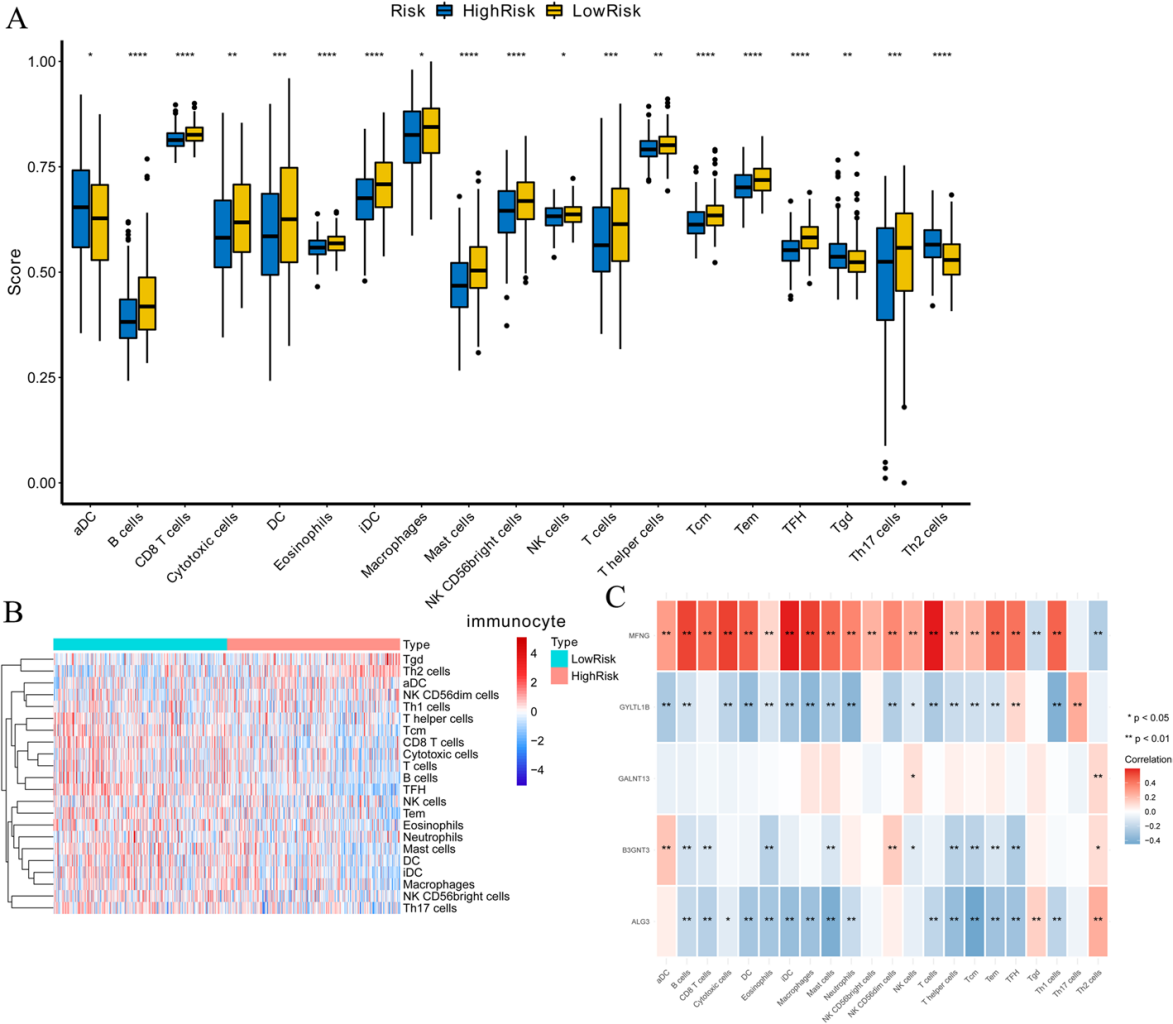
Immune environment analysis of LUAD patients. **A** The infiltration levels of 19 immune cells in high- and low-risk groups calculated by ssGSEA algorithm. **B** The heatmap of the expression level of infiltrating immune cells between high- and low-risk groups. **B** and **C** The correlations between prognostic genes and infiltrating immune cells. * represents p < 0.05, ** represents p < 0.01, *** represents p < 0.001, **** represents p < 0.0001. Red: positive correlation, blue: negative correlation

Cell-mediated immune response and inflammatory response were associated with GTs by IPA analysis, and several studies emphasized the correlation between the immune response and GTs in tumor progressions [8, 9]. Next, we explored the correlation between risk signature and infiltrating immune cells. These results suggested that aDC (high-risk group: 0.647 ± 0.122; low-risk group: 0.620 ± 0.120) and Tgd (high-risk group: 0.543 ± 0.050; low-risk group: 0.530 ± 0.045) were infiltrated in the high-risk group, while B cells (high-risk group: 0.391 ± 0.068; low-risk group: 0.432 ± 0.090), CD8T cells (high-risk group: 0.815 ± 0.023; low-risk group: 0.828 ± 0.023), eosinophils (high-risk group: 0.559 ± 0.025; low-risk group: 0.569 ± 0.026), mast cells (high-risk group: 0.472 ± 0.072; low-risk group: 0.472 ± 0.072), NK CD56 bright cells (high-risk group: 0.640 ± 0.071; low-risk group: 0.665 ± 0.067), and NK cells (high-risk group: 0.630 ± 0.030; low-risk group: 0.636 ± 0.027) had a high infiltrating degree in the low-risk group (Fig. 5A, B). Moreover, the expression of MFNG significantly positively correlated with infiltrating T cells (cor = 0.598, *P* = 7.02E−50), iDC (cor = 0.595, *P* = 3.31E−49), macrophages (cor = 0.543, *P* = 9.99E−40), cytotoxic cells (cor = 0.539, *P* = 5.12E−39), B cells (cor = 0.531, *P* = 7.21E−38), DC (cor = 0.461, *P* = 1.09E−27), Th1 cells (cor = 0.459, *P* = 2.05E−27), and Tem (cor = 0.450, *P* = 2.17E−26). ALG3 expression showed a negative correlation with infiltrating Tcm (cor = −0.449, *P* < 0.01) and mast cells (cor = −0.412, *P* < 0.01) (Fig. 5C). Taken together, our results indicated that the prognostic model provides a novel approach to elucidate the characteristics of immunity regulatory network in LUAD.

### Verification of prognostic model gene expression in LUAD tissue

**Fig. 6:**
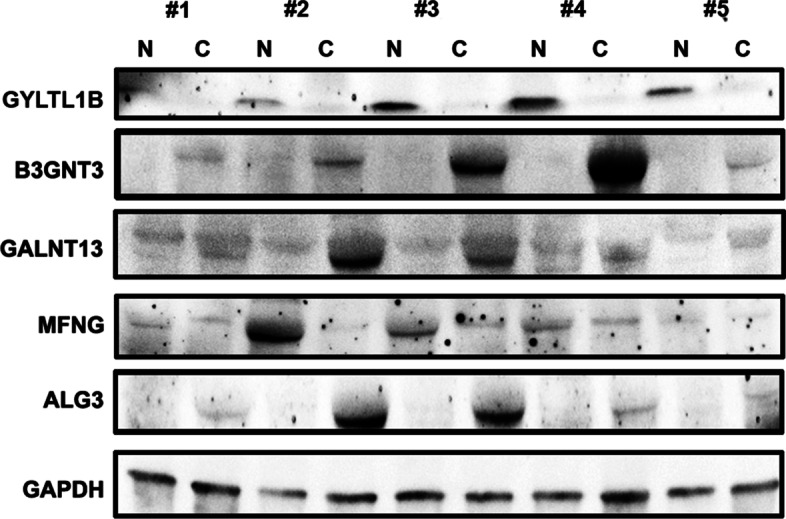
Verification of prognostic model gene expression in LUAD tissues. Western blotting showed that the expression of B3GNT3, ALG3 and GALNT13 in LUAD tissues (C) was higher than that in paracancerous normal tissues (N). The expression of MFNG,GYLTL1B was lower in LUAD tissues

In order to verify the accuracy of our prognostic model, we used western blotting to detect the expression of B3GNT3, MFNG, GYLTL1B, ALG3, and GALNT13 proteins in five pairs of LUAD tissues and paracancerous normal tissues and found that the expression of B3GNT3, ALG3, and GALNT13 in LUAD tissues was significantly higher than that in paracancerous normal tissues, while the expression of MFNG and GYLTL1B was lower in LUAD tissues, which was consistent with the results of our bioinformatics analysis (Fig. 6).

## Discussion

LUAD is the most common subtype of lung cancer, which has a high incidence of malignancy, early metastasis, and a high postoperative recurrence rate, leading to poor prognosis [2]. Although the clinical treatment has been constantly updated in recent years, the 5-year survival rate of LUAD is at a level of 15% [10]. Therefore, searching for new target genes related to the prognosis and targeting the gene to improve the prognosis of LUAD patients is the focus of LUAD research. In this study, we used the bioinformatics methods to screen DEGs in GTs of LUAD patients from TCGA database and confirmed that these genes are closely related to the regulation of protein glycosylation modification, thus affecting the biological behavior of the cells. Interestingly, we also found that the risk score was significantly related to immune cell infiltration.

Abnormal glycosylation of protein is one of the hallmarks of the malignant tumor. Lattová et al. reported significant differences in N-glycan profiles between LUAD and normal tissues adjacent to cancer; also, significant differences were detected among different histologic stages of LUAD, indicating that glycosylation was closely related to the occurrence and development of LUAD [11]. In the present study, we screened out five GTs (B3GNT3, MFNG, GYLTL1B, ALG3, and GALNT13) as an effective approach that might play critical roles in LUAD prognosis. B3GNT3 (beta-1,3-*N*-acetylglucosaminyltransferase-3) forms extended core 1 oligosaccharides by adding GlcNAc to core 1 (T antigen), and its transcripts were expressed in the liver, kidney, pancreas, and other organs [12]. Previous studies have reported a critical role of B3GNT3 in various malignant tumors [12–14]. However, only a few studies were conducted to explore B3GNT3 regulation in LUAD. Leng et al*.* reported a correlation between B3GNT3 and PD-L1/EGFR, which led to a poor prognosis of LUAD [13]. In the present study, we found that B3GNT3 was closely associated with LUAD prognosis. We also confirmed that the expression of B3GNT3 protein in lung adenocarcinoma was significantly higher than in the adjacent normal tissues, consistent with the previous results. However, the molecular biological mechanism of B3GNT3 in LUAD is not yet clarified and needs further exploration. MFNG (manic fringe) is a fucose-specific beta-1, 3-N-acetylglucosaminyltransferase, a member of the fringe family, which mediates the activation of the NOTCH signaling pathway [15]. MFNG is overexpressed in breast cancer [16] and renal cell carcinoma [17] and is associated with poor prognosis. However, different results were found in colorectal cancer [18]. Therefore, MFNG may play a unique role in different cancer types. However, whether MFNG regulates lung cancer tumorigenesis and progression has not been reported. TCGA data analysis found a low expression of MFNG in LUAD, which is related to poor prognosis. Western blotting analysis confirmed that MFNG in LUAD tissues was lower than that in normal tissues adjacent to cancer, which was consistent with the findings of previous studies. These results suggested that MFNG may play an inhibitory role in the occurrence and development of LUAD. GYLTL1B (glycosyltransferase-like 1B) (LARGE2) serves as xylosyltransferase and glucuronyltransferase that can polymerize Xyl-GlcA repeat sequences and mediate the formation of laminin-binding glycans on α-dystroglycan [19]. To date, GYLTL1B has only been shown to be associated with metastasis and prognosis in prostate cancer [19], renal cell carcinoma [20], and colorectal cancer [21]. Dietinger et al. [21] and Huang et al. [22] reported that GYLTL1B expression was regulated by WNT signaling pathway and EMT-related genes; however, its downstream pathway was not clarified. In the present study, for the first time, we reported that the low expression of GYLTL1B in LUAD is related to poor prognosis, which might be caused by the elimination of the functional glycosylation of α-dystrophin. ALG3 (alpha-1,3-mannosyltransferase) is involved in synthesizing N-linked glycans in the early stage, making it a key molecule for N-linked protein glycosylation [23]. Some studies have shown that the overexpression of ALG3 is related to lymph node metastasis in esophageal squamous cell carcinoma [24] and promotes stem cell property and radiation resistance in breast cancer by regulating the glycosylation of TGF-β receptor II [25]. Ke et al*.* confirmed that the expression of ALG3, regulated by miR-98-5p, is significantly increased in non-small cell lung cancer (NSCLC), thus affecting the proliferation, migration, invasion, and poor prognosis [26]. The present study suggested that the expression of ALG3 in LUAD was increased and associated with poor prognosis, which was consistent with the results of other studies. GALNT13 (N-acetylgalactosaminyl transferase 13) encodes the polypeptide N-acetylgalactosaminyl transferase 13 (ppGalNAcT13), a member of the GalNAcT family involved in the initiation of mucin O-link glycosylation [27]. Another study showed that GALNT13 or its product ppGalNAcT13 is abnormally expressed in prostate cancer [27], lung cancer [28], and neuroblastoma [29] and is related to the prognosis of patients. The current results showed an increased expression of GALNT13 in LUAD, which was consistent with that of Nogimori et al. [28]. However, the mechanism of GALNT13 in LUAD is not clear, needing further exploration. In summary, our prognostic model revealed an increased expression of three glycosyltransferases (B3GNT3, ALG3, and GALNT13) and decreased expression of two glycosyltransferases (MFNG and GYLTL1B), which led to the poor prognosis of LUAD. Our prognostic model is more favorable than that of a single gene due to various glycosylation alterations in LUAD. However, the interaction of these genes during glycosylation in LUAD cells needs to be explored further.

The low frequency of anti-tumor infiltrating immune cells indicates impaired immune function and leads to poor prognosis. Our analysis found that B cells, T cells, DCs, and other tumor-infiltrating lymphocytes in the high-risk group were significantly lower than those in the low-risk group, indicating that the immune function of the high-risk group was worse than that of the low-risk group, and the risk score was related to the immune microenvironment of LUAD. In order to further explore how the prognostic model affects the immune function of patients with LUAD, we analyzed each gene in the model individually and found that MFNG is closely related to all the selected immune cells’ infiltration except Th17. Song et al. reported that MFNG was related to the development of T and B cells and was required for optimal in vitro stimulation of T and B cells [30]. MFNG regulated T cell development by enhancing the interaction between Notch1 in T cell progenitor cells and Delta-like 4 in thymic epithelial cells [31]. In the spleen, MFNG co-modified Notch2 in the marginal zone B progenitor cells, enhanced their interaction with Delta-like 1 on endothelial cells, and regulated the production of marginal zone B cells [31]. In addition to B and T cells, we found that MFNG was closely associated with many other immune cells, indicating that it mediates tumorigenesis by regulating the immune response. Du et al. demonstrated that ALG3 is associated with CD8 + T cell infiltration and is closely related to the prognosis of LUAD [32]. Furthermore, ALG3 had a higher correlation with Tcm and mast cells. Tcm [33] and mast cells [34] are closely related to tumorigenesis, suggesting that ALG3 might participate in tumor progression by regulating these immune cells; however, the underlying mechanism needs to be explored further. Among the five genes selected, except for *GALNT13*, which was not significantly related to immune cell infiltration, the other four genes were associated with most immune cell infiltration, and the changes inf the expression level of any one of them would decrease the immune function in LUAD patients, indicating that tumor microenvironment was closely related to protein glycosylation.

Furthermore, our LUAD model had improvements compared to the previous studies. Gu et al. used MRPL51 + SLK + PRC1 to construct the prognostic model; however, the ROC curve showed that the 5-year AUC was < 0.60 [35]. In the present analysis, the AUC values of 1-, 3-, and 5-year training and verification sets were > 0.60, which were reliable. Li et al. identified four lncRNAs and eight mRNAs as prognostic markers for LUAD to construct prognostic models without using internal and external validation sets [36]. We screened out five markers to construct the prognostic models as well as internal and external validation sets, verified via line maps. Despite these advantages, the present study has some limitations. Firstly, this study is based on the retrospective analysis of bioinformatics based on the TCGA database. Secondly, although we used GEO database to verify the prognosis model, due to the small overall sample size, a large amount of experimental and clinical data are still needed. Thirdly, differential genes were screened by R package, and Cox regression was used to establish a prognostic model. Thus, in our future study, other methods will be used to screen out the genes as described previously [37–39]. In addition, in vivo and in vitro studies are needed to explore the biological role of glycosylation-related genes in LUAD and support our findings.

## Conclusions

In conclusion, we screened five glycosylation-related genes (*B3GNT3*, *MFNG*, *GYLTL1B*, *ALG3*, and *GALNT13*) that were closely related to the prognosis of LUAD patients by bioinformatics methods, established a new risk score model, and verified that the risk score was closely related to the prognosis of LUAD. Furthermore, we found that the risk score was significantly associated with immune cell infiltration. However, the biological role of glycosylation-related genes in LUAD needs to be explored further.

## Materials and methods

### Data resource

The gene expression profile of 519 LUAD samples and 58 control samples from the TCGA database (https://cancergenome.nih.gov/) were acquired. Moreover, 210 GTs from the GlycomeDB database (www.glycome-db.org) that provided the structural and taxonomic information of all major public carbohydrates were downloaded. GSE31210 dataset contained 226 LAUD samples, and GSE72094 dataset with 442 LUAD samples was used as a validated set.

### Differentially expressed analysis

The transcriptomes of 519 LUAD samples and 58 control samples from the TCGA database were used to identify DEGs using edge R package. The threshold parameters were as follows: |Log_2_ fold change |≥ 1 and *P*-value < 0.05. Then, the screened DEGs were intersected with 210 GTs, downloaded from the GlycomeDB database to obtain the DGTs. The correlations among these DGTs were explored by Pearson analysis using the R package coreeplot.

### Functional enrichment analysis

The functional enrichment analysis of the DGTs was carried out by GO and KEGG (www.kegg.jp/kegg/kegg1.html) [40–42] analyses using clusterProfiler package in R. IPA database (http://www.ingenuity.com/products/ipa) was adopted to investigate the canonical pathways and top network of the DGTs. *P*-value < 0.05 was set as the statistically significant cutoff in these analyses.

### Construction of a prognostic model

501 LUAD samples and correspond survival data in the TCGA-LUAD cohort were survival as a training set. Subsequently, univariate Cox regression analysis screened the GTs-related genes that were significantly associated with the prognosis of LUAD with *P*-value < 0.05 as a selection criterion in the training set. The screened genes were further analyzed by multivariate Cox regression analysis and stepwise analysis to identify the candidate prognostic genes to construct a prognostic risk model for LUAD. We determined the risk score of each LUAD patient using the following formula:

$${\text{Risk\,score}} ={\text{e}}^{{\text{sum(each\,gene's\,expression\,levels}}} \times^{{{\text{corresponding\,coefficient)}}}} /{\text{e}}^{{{\text{sum(each\,gene's\,mean\,expression\,levels}}}} \times^{{{\text{corresponding\,coefficient)}}}}$$

We distinguished the patients in the training set with a high-risk score from those with a low-risk score based on the median value of the risk score. The different OS between the two risk groups was detected by K–M survival analysis using the survival package in R. Moreover, the ROC curve analysis was applied to assess the efficiency and accuracy of the established prognostic risk model of LUAD with the R package survival ROC. 226 LUAD patients in the GSE31210 dataset and 422 LUAD samples in the GSE72094 dataset were utilized to verify the specificity and sensitivity of the prognostic model.

### Establishment of a nomogram

Univariate and multivariate Cox regression analyses were conducted on the clinical characteristics (age, gender, stage, T, M, and N) and risk score to explore the prognostic value of these factors using the survival R package. The independent prognostic factors were included to construct a nomogram using R language rms to predict the survival probability of 1-, 3-, and 5-years for LUAD patients in the training set. A calibration curve plot was performed to evaluate the nomogram performance.

### Immune microenvironment analysis

ssGSEA algorithm was utilized to evaluate the immune score of each LUAD sample from the TCGA database using R package gsea. The different proportions of the infiltrating immune cells between the two risk groups were assessed by Wilcoxon test. Spearman’s analysis was employed to explore the correlations among the prognostic genes in the risk model and the 22 infiltrating immune cell types.

### LUAD patient samples

LUAD tissues and paracancerous normal tissues were collected from 5 patients during surgery at the Zhejiang Cancer Hospital in 2021. All patients did not receive any treatment before the operation, and the pathology was confirmed as LUAD. This study was approved by the ethics committee of Zhejiang Cancer Hospital. All participants provided informed consent for sample collection, intended research, and publication usage. Written consent was collected according to the ethical regulations of Zhejiang Cancer Hospital.

### Western blotting

The tissue samples were homogenized with liquid nitrogen and lysed using RIPA [25 mM Tris–HCl (pH 7.6), 150 mM NaCl, 1% NP-40, 1% sodium deoxycholate, 0.1% SDS] with protease and phosphatase inhibitors. After sonication for 2 min at 4 ℃ and centrifugation for 10 min at 4 ℃, the supernatant was considered as the total cell lysate. An equivalent of 50 µg total protein was analyzed by SDS-PAGE and transferred to the nitrocellulose (NC) membrane. Subsequently, the blot was probed with primary antibodies [B3GNT3 (Proteintech, cat. no. 18098-1-AP), MFNG (Affinity, cat. no. DF3837), GYLTL1B (Atlas, cat. no. HPA056621), ALG3 (Proteintech, cat. no. 20290-1-AP), GALNT13 (Bioss, cat. no. bs-13274R), and GAPDH (Proteintech, cat. no. 10494-1-AP)] and then incubated with horseradish peroxidase (HRP)-labeled secondary antibodies for visualization using enhanced chemiluminescence reagents (Thermo). The images were obtained on a Bio-Rad Imaging System.

### Statistical analysis

The box and volcano plots in our study were drawn using the ggplot2 package in R. The correlation between the risk score and clinical characteristics was analyzed by the Wilcox test; *P* < 0.05 indicated a statistically significant difference.

## Supplementary Information

**Additional file 1.** Original data and codes related to this article.

**Additional file 2.** Original data of western blotting.

**Additional file 3. Fig. S1**: Validation of the risk model with the GSE72094 dataset. (A) KM survival curve of OS of LUAD patients. (B) ROC curve analysis for measuring the prognostic performance of the signature-based risk score on OS. (C) Risk score, survival status, and 5 GTs expression heatmap of the LUAD patients.

**Additional file 4. Table S1. **The differentially expressed analysis with 519 LUAD samples and 58 control samples from the TCGA database.

## Data Availability

All data generated or analyzed during this study are included in this published article and its supplementary information files.
