# Supplementary figures and images for: A novel glycosylation-related gene signature predicts survival in patients with lung adenocarcinoma
Source: BMC Bioinformatics. 2022 Dec 27;23:562. doi: 10.1186/s12859-022-05109-8 (PMC9793550; doi:10.1186/s12859-022-05109-8)

ALG3


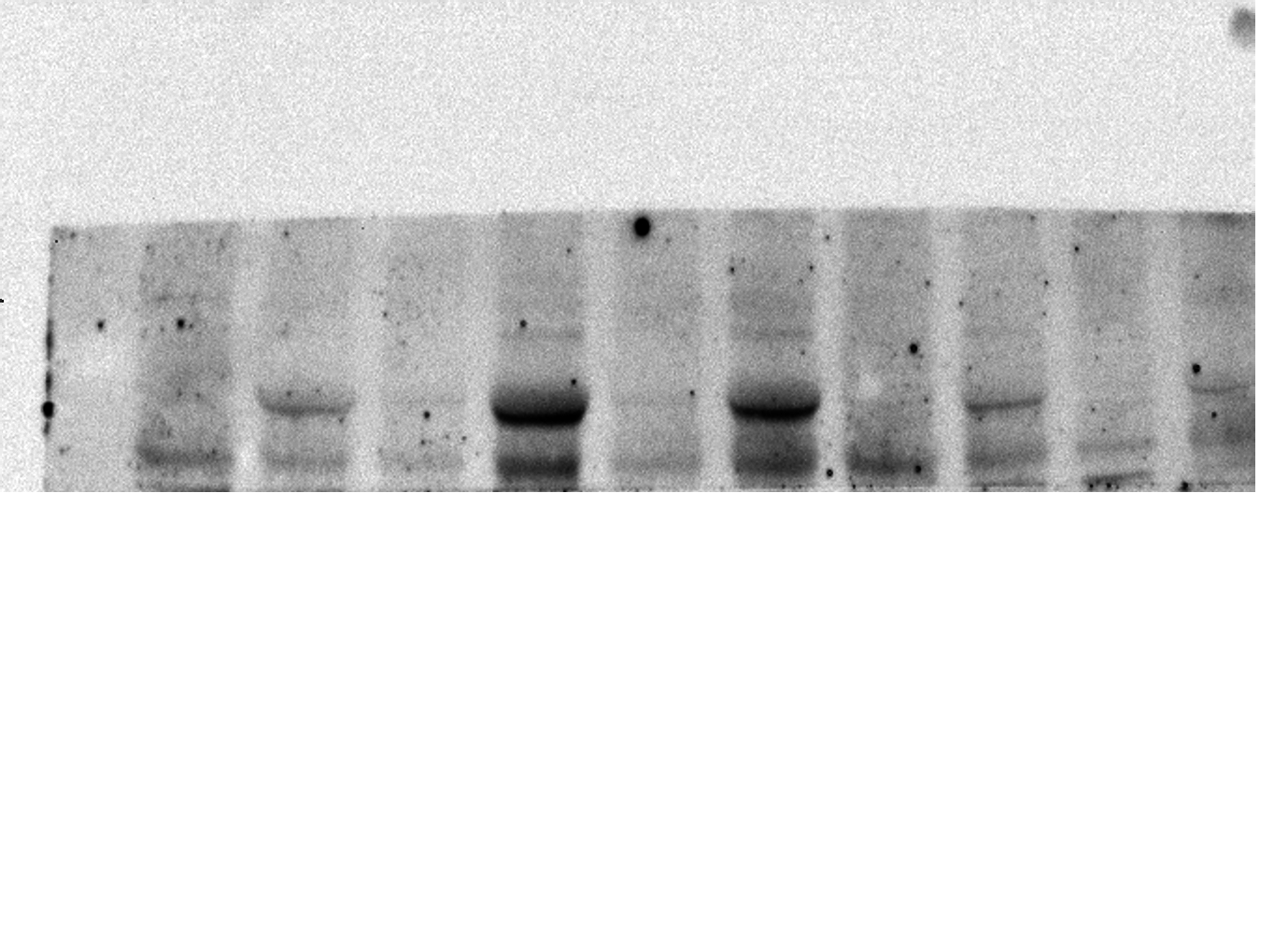


B3GNT3


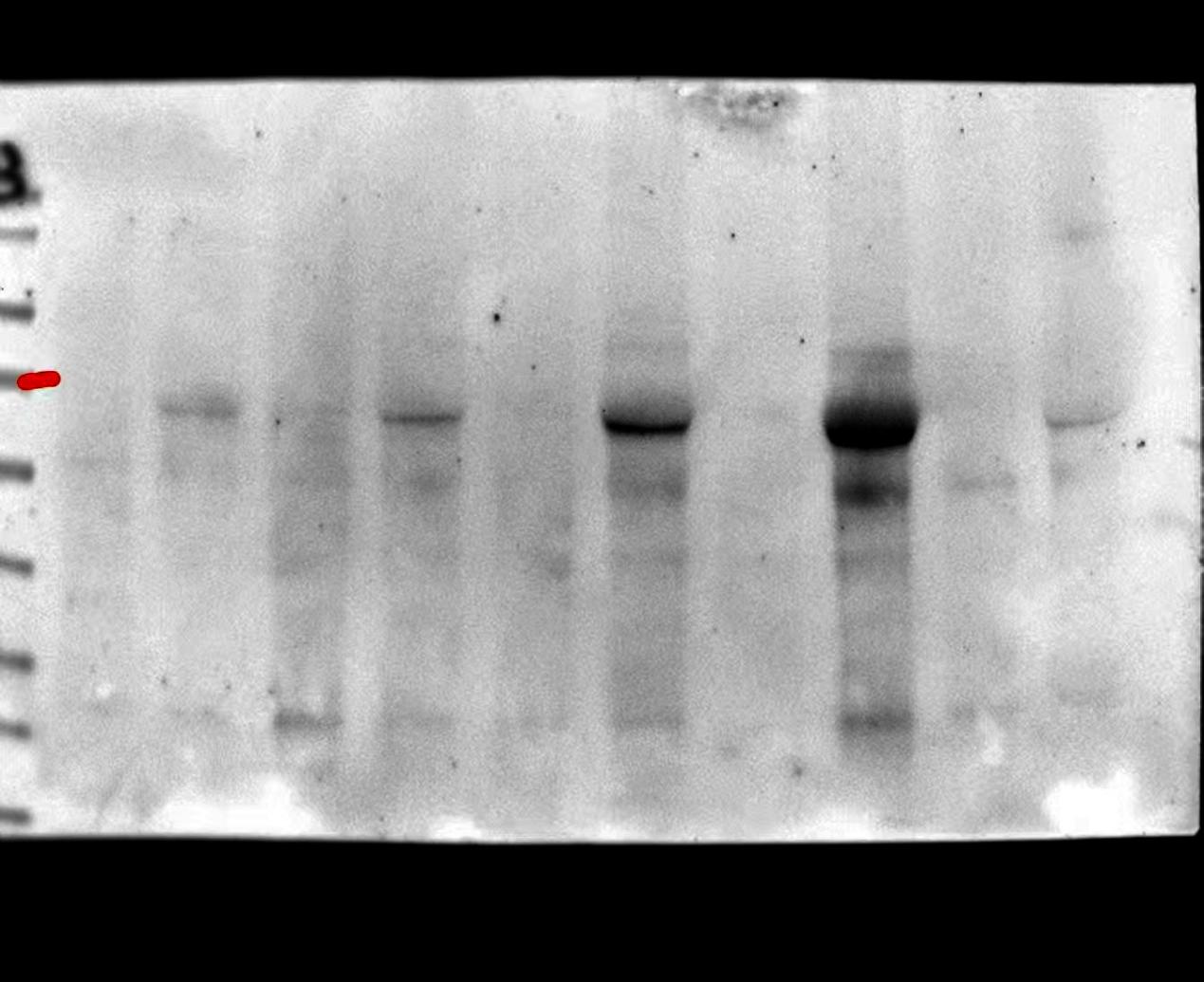


GALNT13


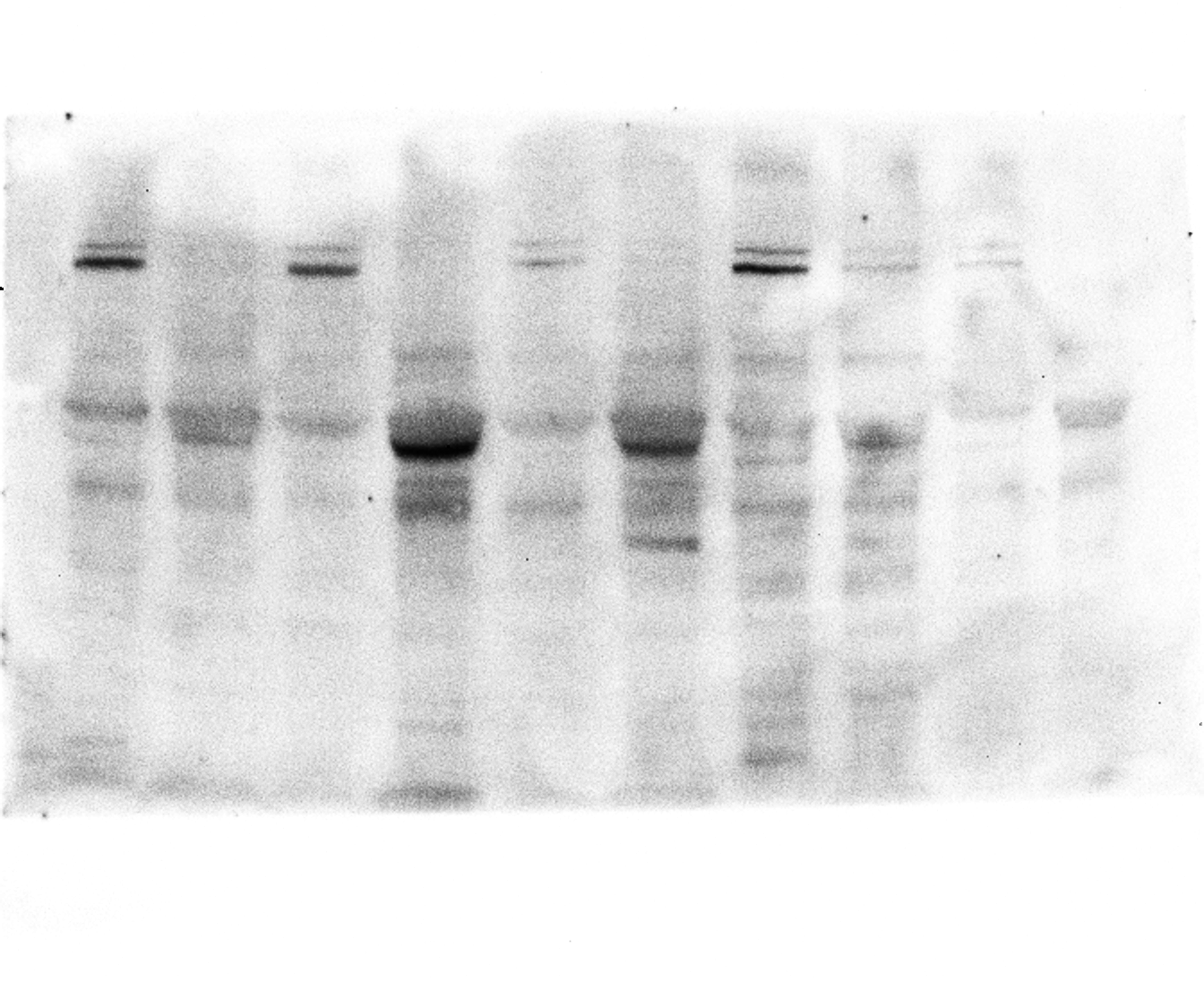


GYLTL1B


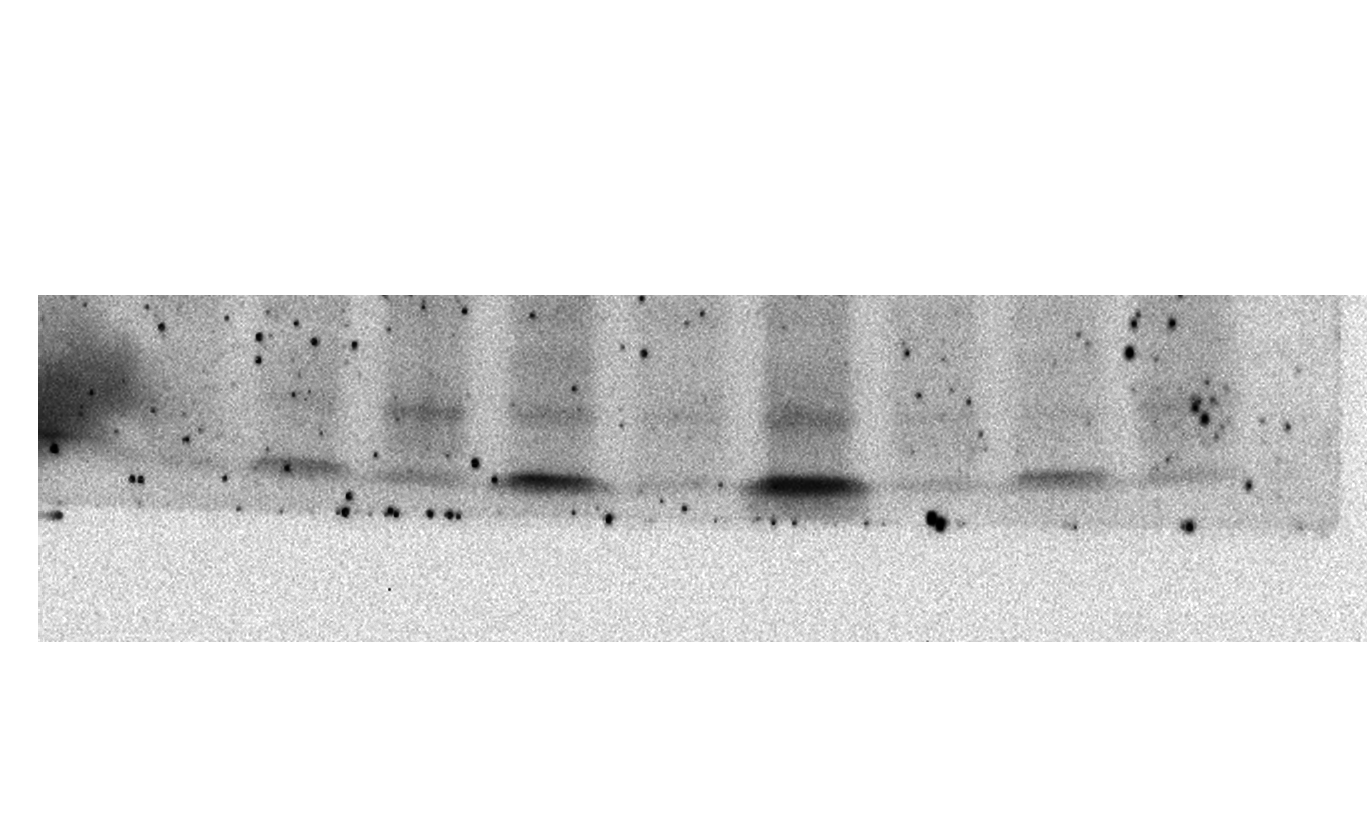


MFNG


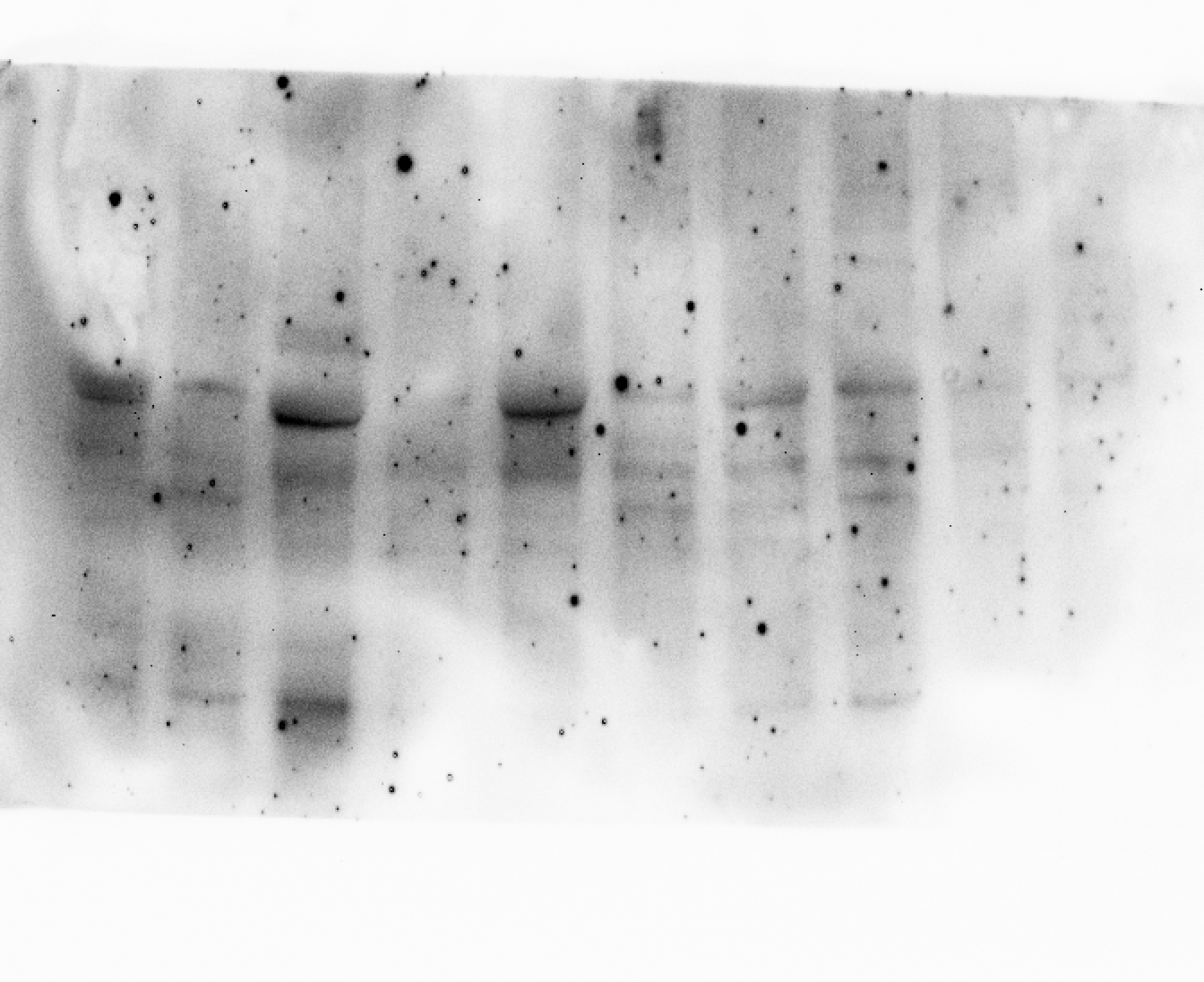


GAPDH


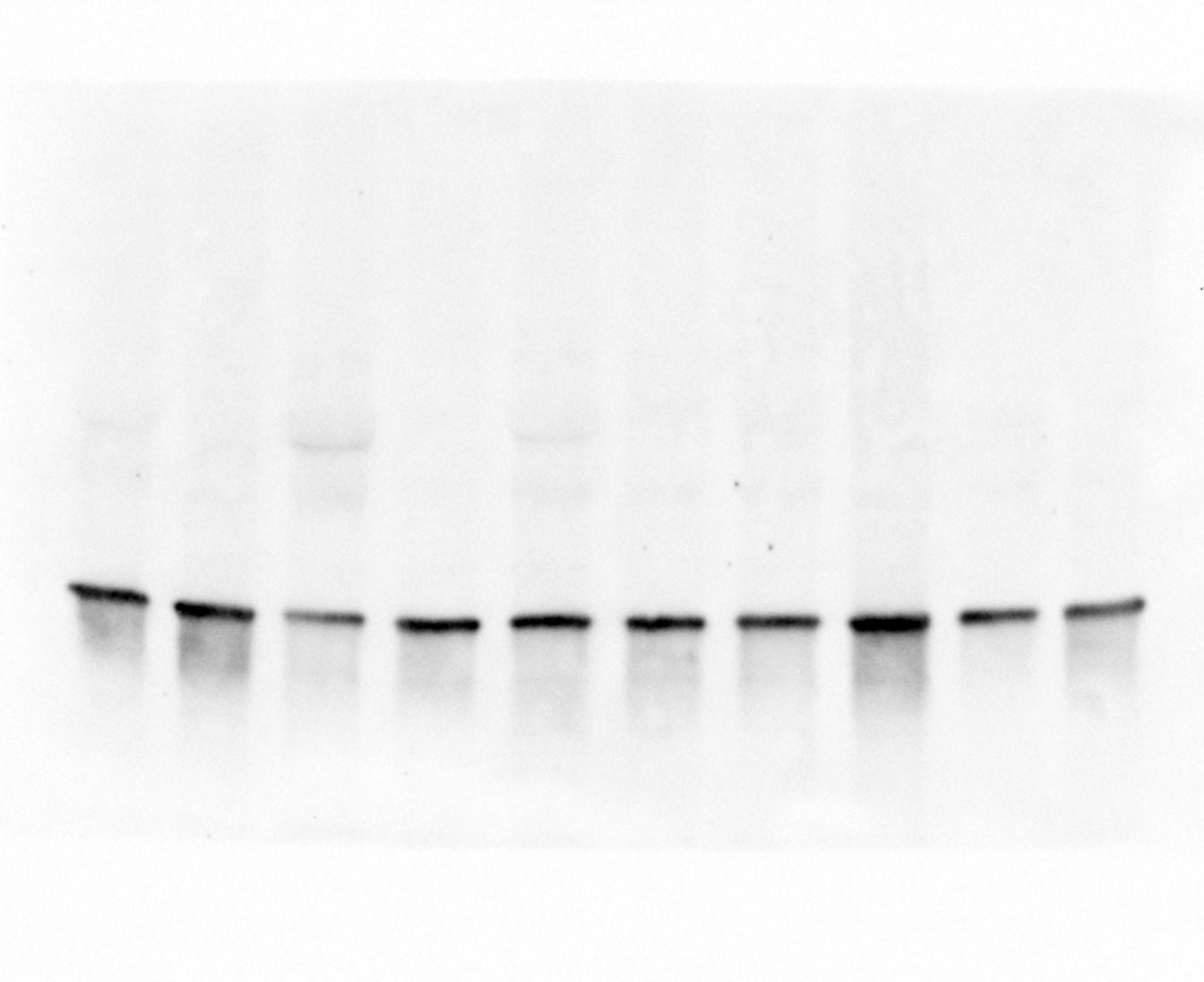

Supplement: Supplementary file 2 — Additional file 2. Original data of western blotting. [file 12859_2022_5109_MOESM2_ESM.docx]

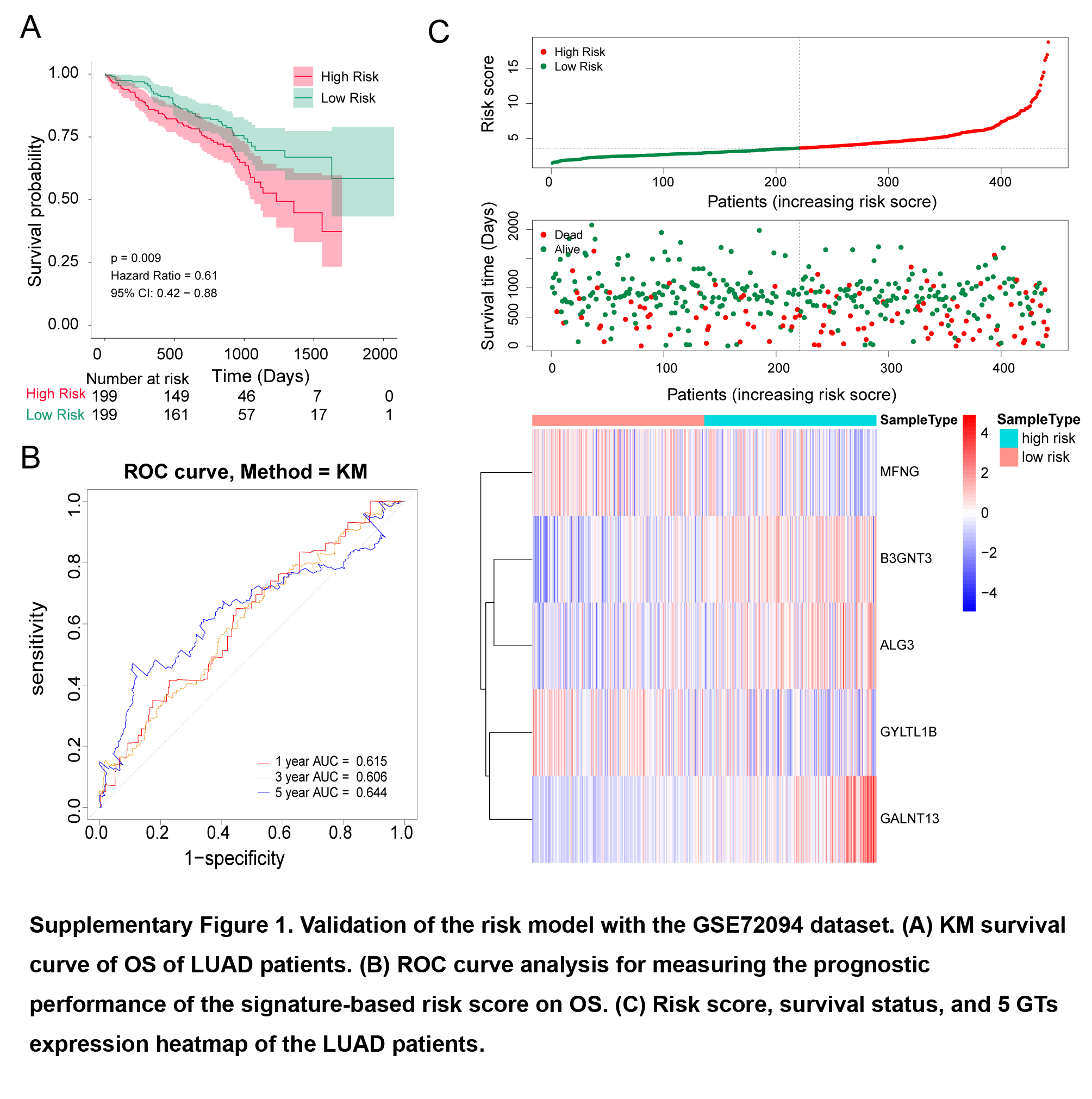

Supplement: Supplementary file 3 — Additional file 3. Fig. S1: Validation of the risk model with the GSE72094 dataset. (A) KM survival curve of OS of LUAD patients. (B) ROC curve analysis for measuring the prognostic performance of the signature-based risk score on OS. (C) Risk score, survival status, and 5 GTs expression heatmap of the LUAD patients. [file 12859_2022_5109_MOESM3_ESM.tif]
